# Development and application of deep learning-based diagnostics for pathologic diagnosis of gastric endoscopic submucosal dissection specimens

**DOI:** 10.1007/s10120-025-01612-y

**Published:** 2025-04-15

**Authors:** Soomin Ahn, Yiyu Hong, Sujin Park, Yunjoo Cho, Inwoo Hwang, Ji Min Na, Hyuk Lee, Byung-Hoon Min, Jun Haeng Lee, Jae J. Kim, Kyoung-Mee Kim

**Affiliations:** 1 Department of Pathology and Translational Genomics, Samsung Medical Center, Sungkyunkwan University School of Medicine, Seoul, South Korea; 2 Department of R&D Center, Arontier Co., Ltd, Seoul, South Korea; 3 Department of Internal Medicine, Samsung Medical Center, Sungkyunkwan University School of Medicine, Seoul, South Korea

**Keywords:** Gastric cancer, Endoscopic submucosal dissection, Deep learning, Diagnosis

## Abstract

**Background:**

Accurate diagnosis of ESD specimens is crucial for managing early gastric cancer. Identifying tumor areas in serially sectioned ESD specimens requires experience and is time-consuming. This study aimed to develop and evaluate a deep learning model for diagnosing ESD specimens.

**Methods:**

Whole-slide images of 366 ESD specimens of adenocarcinoma were analyzed, with 2257 annotated regions of interest (tumor and muscularis mucosa) and 83,839 patch images. The development set was divided into training and internal validation sets. Tissue segmentation performance was evaluated using the internal validation set. A detection algorithm for tumor and submucosal invasion at the whole-slide image level was developed, and its performance was evaluated using a test set.

**Results:**

The model achieved Dice coefficients of 0.85 and 0.79 for segmentation of tumor and muscularis mucosa, respectively. In the test set, the diagnostic performance of tumor detection, measured by the AUROC, was 0.995, with a specificity of 1.000 and a sensitivity of 0.947. For detecting submucosal invasion, the model achieved an AUROC of 0.981, with a specificity of 0.956 and a sensitivity of 0.907. Pathologists’ performance in diagnosing ESD specimens was evaluated with and without assistance from the deep learning model, and the model significantly reduced the mean diagnosis time (747 s without assistance vs. 478 s with assistance, *P* < 0.001).

**Conclusion:**

The deep learning model demonstrated satisfactory performance in tissue segmentation and high accuracy in detecting tumors and submucosal invasion. This model can potentially serve as a screening tool in the histopathological diagnosis of ESD specimens.

## Introduction

Gastric cancer is the fifth most common malignant tumor worldwide, with approximately one million new cases annually [1]. Among gastric cancers, the proportion with early gastric cancer (EGC) has been increasing, particularly in East Asia, owing to early detection through screening programs. Endoscopic submucosal dissection (ESD) is the standard treatment for EGC with low risk of lymph node metastasis [2, 3]. The histopathological diagnosis of ESD specimens is critical for decision-making for further treatment. Based on the histopathological diagnosis, patients at risk of lymph node metastasis or non-curative resection undergo gastrectomy with lymph node dissection. Therefore, the accurate histopathological diagnosis of ESD specimens depends on assessment of all risk factors, including the depth of invasion, lymphatic invasion, tumor size, and margin status [2].

Microscopic evaluation based on hematoxylin and eosin (H&E)-stained slides is the gold standard for histopathological diagnosis. However, histopathology departments face several challenges, including an increase in the volume of specimens, a shortage of pathologists, inter-observer discordance, and inherent human errors [4, 5]. The histopathological diagnosis of ESD specimens is a multi-step, time-consuming process that requires not only identification of the tumor but also evaluation of multiple factors. In addition, accurate diagnosis in serially cut ESD specimens requires experience, because small foci of submucosal invasion or lymphatic emboli can be missed. Consequently, there is a demand for support systems for the histopathological diagnosis of ESD specimens.

In recent years, digital pathology has emerged as a powerful tool for analyzing histopathological specimens. The application of artificial intelligence (AI) to digitized whole-slide images (WSIs) is a promising solution to assist in histopathological diagnosis. There have been some studies on an AI-based histopathological diagnosis of gastric cancer, mostly for biopsy specimens [6–12]. However, no AI-based histopathological diagnosis has been attempted for ESD specimens of EGC. In this study, we aimed to develop and evaluate a deep learning model for the diagnosis of ESD specimens of EGC.

## Materials and methods

### Study design

**Fig. 1:**
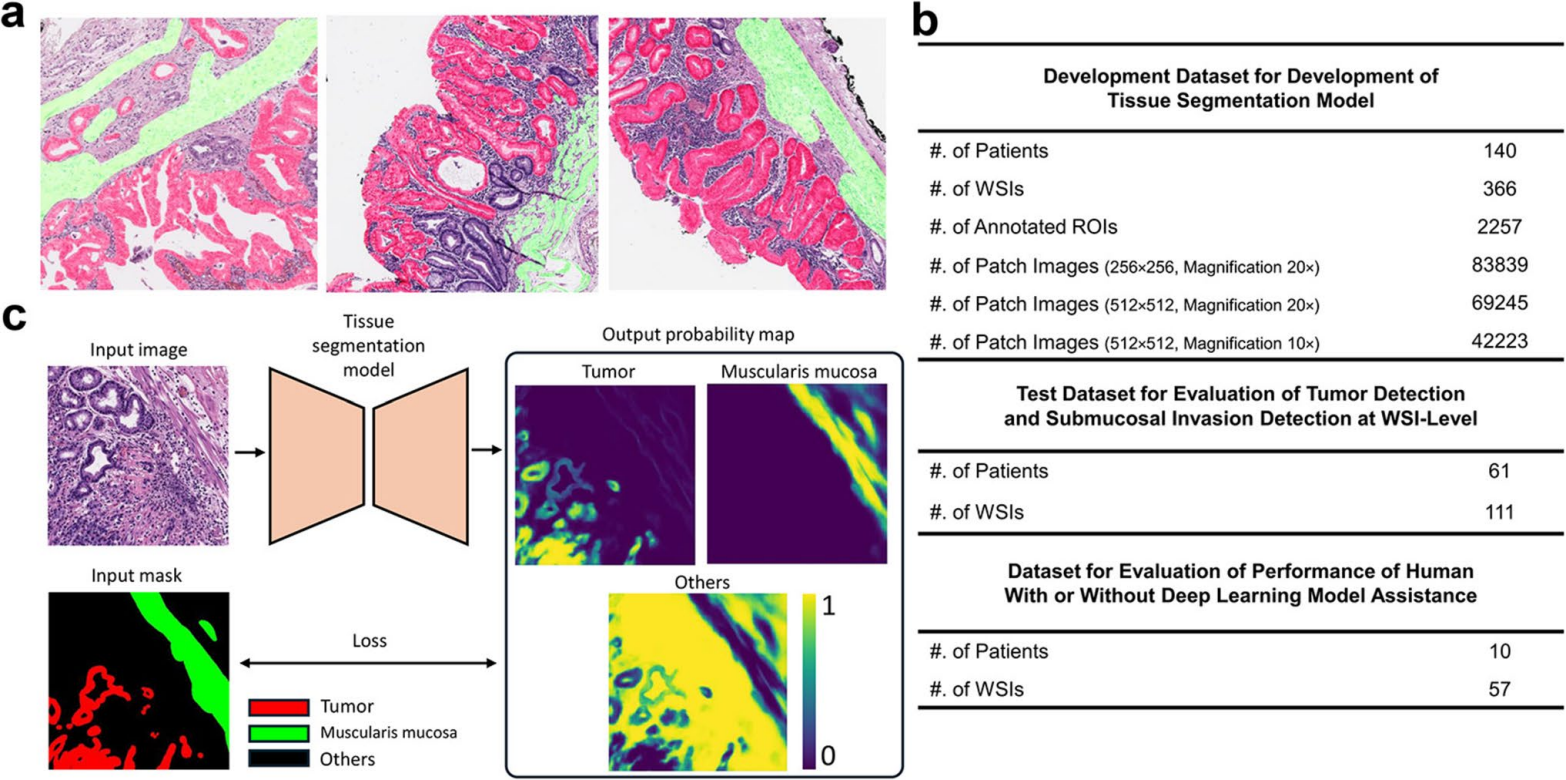
Overview of the data and deep learning framework for tissue segmentation presented in this study. (**a**) Sample of training images with annotation for developing tissue segmentation model. Red annotation represents tumor, green annotation represents muscularis mucosa. (**b**) Description of dataset for tissue segmentation model development and dataset for WSI-level tumor detection evaluation and submucosal invasion detection evaluation. (**c**) Deep learning framework for tissue segmentation model. Feeding H&E image to tissue segmentation model, it will output three channel probability maps, each for tumor, muscularis mucosa and others class, the loss between the output channels and the ground truth input mask is backpropagated to the model to update its weights to conduct training process

**Fig. 2:**
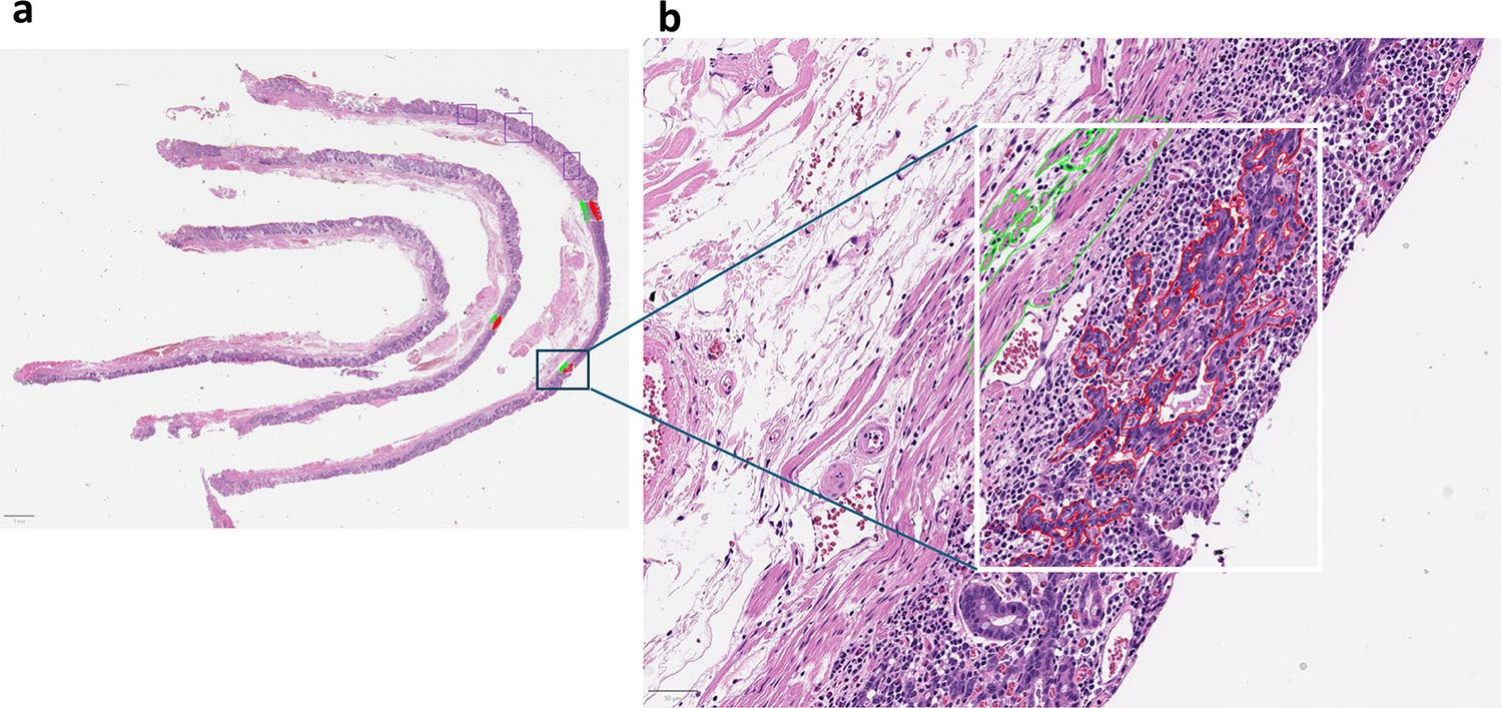
An example of manually annotated tumor region. (**a**) A total of 2257 regions of interest is marked, and (**b**) pixel-level annotation is performed for the tumor (red) and muscularis mucosa (green) by a gastrointestinal pathologist

An overview of this study is presented in Fig. 1. The dataset consisted of a development set and a test set. To develop a tissue segmentation model, 366 WSIs of ESD from 140 patients with EGC were retrospectively enrolled. The scanned WSIs were manually annotated using QuPath v0.2.0-m2 software [13] by S.A., a gastrointestinal histopathologist with 12 years of experience. For each WSI, two to 10 regions of interest, approximately 0.5-mm^2^ size, were selected (Fig. 2a). Most tumor areas and some non-tumor areas were included. A total of 2257 regions of interest were marked, and pixel-level annotation was performed for the tumor and muscularis mucosa (Fig. 2b) generating 83,839 patch images. An example of manually annotated tumor region is shown in Fig. 2. The development dataset was randomly divided in a 5:1 ratio into a training set and an internal validation set, and the tissue segmentation performance at the patch level was evaluated in the internal validation set.

**Fig. 3:**
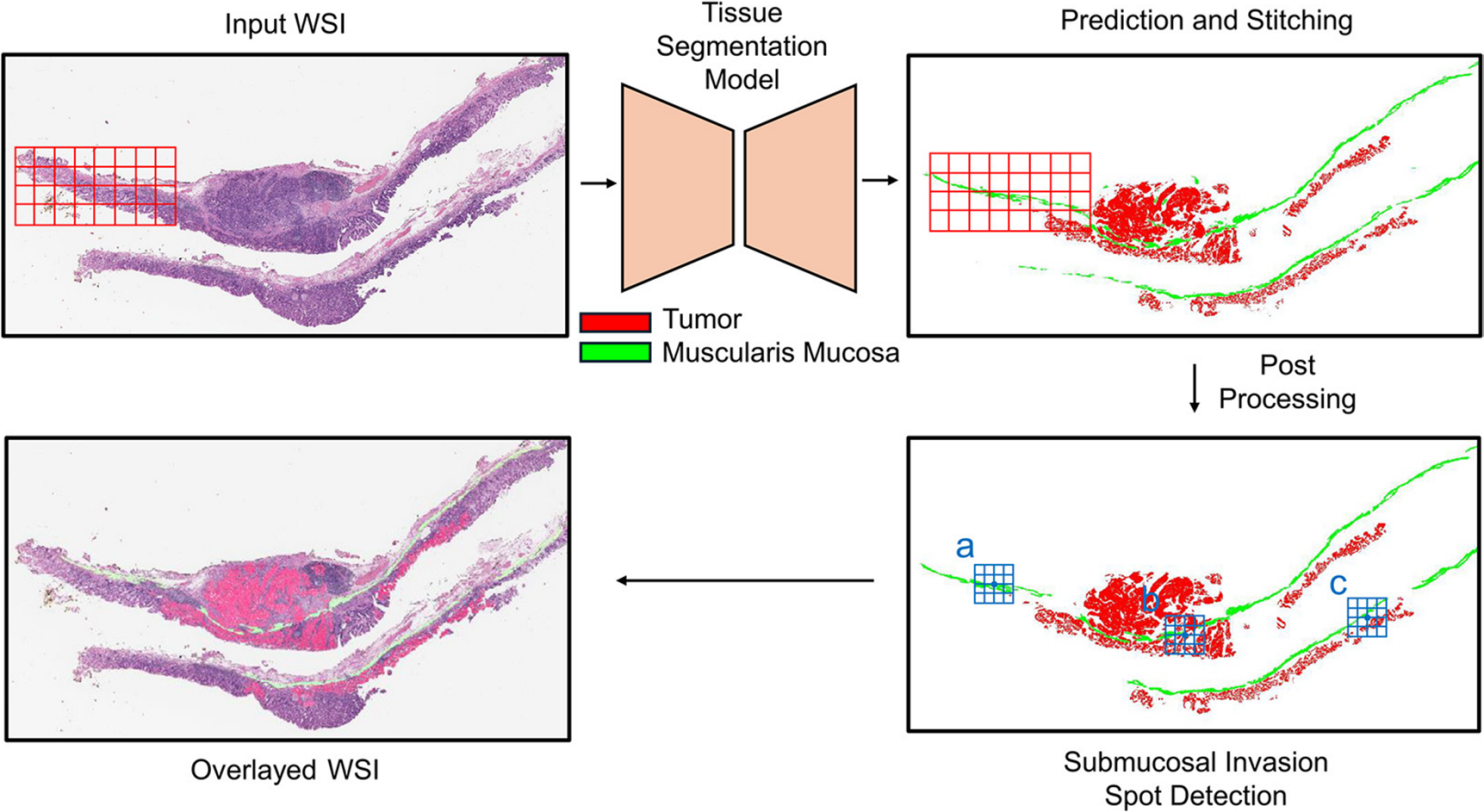
Pipeline for WSI-level tumor detection and submucosal invasion detection. H&E WSI is first tiled into patches and input to the trained tissue segmentation model to predict tumor and muscularis mucosa. The output patches are then stitched together to generate WSI-level tissue segmentation. If there is tumor segment in the output WSI, it is considered tumor is detected. For submucosal invasion detection, we draw boxes with grids along the detected muscularis mucosa, if a lot of grids in a box is filled with tumor, it is considered as a submucosal invasion spot. The final overlayed WSI shows predicted tumor and muscularis mucosa overlayed on the H&E WSI

Next, a detection algorithm for tumor and submucosal invasion at the WSI level was developed (Fig. 3). Its performance was tested using another test dataset comprising 111 WSIs from 61 patients. It consisted of 76 tumor WSIs and 35 non-tumor WSIs. The presence of tumor and submucosal invasion in each WSI was annotated using QuPath v0.2.0-m2 software [13] by S.A., and the prediction of tumor and submucosal invasion by the algorithm was compared with human annotation. This study was approved by the Institutional Review Board of Samsung Medical Center (IRB no. 2021-02-146). The requirement for informed consent was waived by the institutional review board because the samples were anonymized.

### Dataset

The development and test cohorts were obtained from the Samsung Medical Center from ESD specimens received between January 2020 and June 2024. The ESD specimens were cut at 2-mm intervals and serially embedded. The number of sections per slide was typically three (range: 1–4). The average number of slides per patient varied from 3 to 26. In the development cohort, representative slides from each patient were selected. For the test cohort, either representative slides from the patients or all slides from some patients were used. The H&E slides were scanned using an APERIO AT2 (Vista, CA, USA) at 20 × magnification. All cases were histologically diagnosed as adenocarcinoma, and cases with pre-malignant lesions or other tumor types, such as neuroendocrine tumors, were excluded. The clinicopathological information from the dataset is presented in Table 1.

**Table 1:** Clinicopathologic information of dataset

	Development dataset—for development of tissue segmentation model (*n* = 140)	Test dataset—for submucosal invasion detection at WSI-level (*n* = 61)	Dataset for evaluation of pathologists' performance (*n* = 10)^a^
Sex			
Male	97 (69.3%)	43 (70.5%)	8 (80%)
Female	43 (30.7%)	18 (29.5%)	2 (20%)
Age	64.6 (SD 9.7)	69.1 (SD 8.0)	70.8 (SD 6.4)
Size	17.1 (SD 9.8)	18.6 (SD 9.1)	14 (SD 5.6)
Histology by WHO classification^b^			
Tubular adenocarcioma, well differentiated	48 (34.3%)	12 (19.7%)	4 (40%)
Tubular adenocarcioma, moderately differentiated	74 (52.9%)	41 (67.2%)	6 (60%)
Tubular adenocarcioma, poorly differentiated	1 (0.7%)	1 (1.6%)	0 (0%)
Poorly cohesive carcinoma	14 (10.0%)	1 (1.6%)	0 (0%)
Others^c^	3 (2.1%)	6 (9.8%)	0 (0%)
Any poorly differentiated component			
Present	26 (18.6%)	10 (16.4%)	0 (0%)
Absent	114 (81.4%)	51 (83.6%)	10 (100%)
Lauren classification			
Intestinal	115 (82.1%)	56 (91.8%)	10 (100%)
Diffuse	11 (7.9%)	1 (1.6%)	0 (0%)
Mixed	12 (8.6%)	1 (1.6%)	0 (0%)
Indeterminate	2 (1.4%)	3 (4.9%)	0 (0%)
Depth of invasion			
Mucosa	95 (67.9%)	18 (29.5%)	3 (30%)
Submucosa	45 (32.1%)	43 (70.5%)	7 (70%)
Lymphatic invasion			
Present	23 (16.4%)	23 (37.7%)	2 (20%)
Absent	117 (83.6%)	38 (62.3%)	8 (80%)
Resection margin			
Free from tumor	130 (92.9%)	52 (85.2%)	9 (90%)
Involvement of tumor	10 (7.1%)	9 (14.8%)	1 (10%)

*WSI* whole-slide image, *SD* standard deviation

^a^Ten cases were selected from test dataset

^b^In case of mixed histology, dominant histology was chosen

^c^Others include papillary adenocarcinomas, adenocarcinomas with lymphoid stroma, and mucinous carcinomas

The cases in the development set were randomly selected during the study period. Based on the Lauren classification, 82.1% were classified as intestinal type, and 7.9% as diffuse type. Since gastric cancers often exhibit heterogeneous histologic features, each WSI was reviewed for subcomponents. Among the 140 cases, poorly differentiated components (≥ 5%) were identified in 18.6%. There was mucosal cancer in 67.9% and submucosal invasion in 32.1%. The test set was intentionally enriched with tumors exhibiting submucosal invasion (70.5%). According to the Lauren classification, 91.8% were classified as intestinal type, and 1.6% as diffuse type. Poorly differentiated components (≥ 5%) were identified in 16.4%. Finally, slides from 10 patients were selected from the test dataset to evaluate the performance of the pathologists. There were three mucosal cancers, and seven with submucosal invasion. The three mucosal cancers were histopathologically well-differentiated tubular gastric foveolar adenocarcinomas.

### Tissue segmentation model and training details

Three widely used semantic segmentation models were selected (U-Net++ [14], DeepLabv3+ [15], and SegFormer [16]) to segment the gastric tissue H&E images into three classes: tumor, muscularis mucosa, and others. U-Net++ and DeepLabV3+ are convolutional neural network-based models, for which EfficientNet-B3 [17] was used as the backbone and SegFormer, a transformer-based model used MiT-B1 as its backbone. These models were implemented using the PyTorch framework [18] and Segmentation Models Pytorch library [19]. They were trained and evaluated on the patch-level dataset (Fig. 1b**)** to identify the best-performing model for further testing on detection of tumor and submucosal invasion at the WSI level.

The model-training process is illustrated in Fig. 1c. An H&E-stained image is fed into the tissue segmentation model to produce a three-channel probability map of the same size, with each channel representing a specific class. The model iteratively improves predictions by minimizing the loss between the output probability map and the input mask, which corresponds to the ground truth segmentation derived from human annotation. The pixel-wise categorical cross-entropy loss function is defined as:

1 $$\text{loss}=-\sum_{i}^{H}\sum_{j}^{W}\sum_{c}^{C}{y}_{ijc}\text{log}{\widehat{y}}_{ijc}$$

where $H$ and $W$ are the height and width of the image, $C$ is the number of classes, ${y}_{ijc}$ is a binary indicator (0 or 1) if the class label *c* is the correct classification for pixel (*i*, *j*), ${\widehat{y}}_{ijc}$ is the predicted probability that pixel (*i*, *j*) belongs to class *c*.

For model training, the RAdam optimizer [20] was used with a learning rate of 0.0001. The learning rate was reduced by 0.5 every 30 epochs, with a batch size of 32. Training of the models was stopped when there was no improvement in the validation loss. Common image augmentation techniques, such as blurring, rotation, flipping, color jitter, and cutmix [21] were applied to enhance the robustness and generalization ability of the model.

### Submucosal invasion detection algorithm

The process of detecting submucosal invasion at the WSI level is shown in Fig. 3. First, the WSI was divided into patches matching the size used to train the tissue segmentation model. These patches were then inputted into the model to predict tumor and muscularis mucosa at the pixel level. To conserve memory and computational resources during post-processing, the predicted patch maps were scaled down and reassembled into a complete WSI-level map.

The algorithm for detecting submucosal invasion using the identified tumor and muscularis mucosa regions is described below. Submucosal invasion occurs when a tumor breaks through the muscularis mucosa and infiltrates the submucosa. The detection method focused on the extent to which the tumor is distributed within the muscularis mucosa. Specifically, if tumor is present in a substantial portion of the surrounding area at a given point along the muscularis mucosa, it is inferred that there is a high likelihood of submucosal invasion. The bottom-right image in Fig. 3 illustrates the concept of this algorithm. Three points are centered on the detected muscularis mucosa pixels, with a square grid representing the surrounding area. Spot “a” has no tumor around it; therefore, it cannot be an invasion spot. Spots “b” and “c” have tumor in the grids, but spot “b” has a higher proportion of grids with tumor compared to spot “c”. Therefore, spot “b” is more likely to be a submucosal invasion spot. According to the algorithm, if more than 50% of the grids around a spot have tumor, it is likely to be a submucosal invasion spot.

The detailed submucosal invasion detection algorithm is as follows. For each point on the detected muscularis mucosa, an $n\times n$ grid is centered on that point. For each grid, we count the number of squares, m, where a tumor is detected. A higher $m$ indicates a higher probability of submucosal invasion. The cutoff of $m$, was defined as *δ*, and $p$ was defined as the probability that a WSI contains submucosal invasion. Two approaches were used to calculate $p$:

1. Max-based approach: The maximum $m$ value in the WSI, denoted by ${m}_{max}$, is used. The $p$ is then calculated as:
  2 $$p = \begin{cases}\displaystyle \frac{m_{\max}}{n^2}, & \text{if } m_{\max} > \delta, \\0, & \text{if } m_{\max} \leq \delta.\end{cases}$$
2. Mean-based approach: If there are $k$ grids where ${m}_{i}>\delta$, the probability $p$ is calculated as:
  3 $$p = \begin{cases}\displaystyle \left( \sum_i^k \frac{m_i}{n^2} \right)\! \bigg/ k, & \text{if } k > 0, \\0, & \text{if } k = 0.\end{cases}$$

### Application of the deep learning model in histopathological diagnosis

The performance of the pathologists in the diagnosis using WSIs of slides from ESDs with and without deep learning model assistance was evaluated. Three pathologists (YJ. C., I. H, and JM. N) evaluated 57 digitally scanned WSIs of 10 ESDs from the test dataset and were requested to mark the tumor area and complete the diagnostic format used in routine diagnostic practice. With the assistance of the deep learning model, tissue segmentation was provided; a heat-map flagging tumor and muscularis mucosa over the WSI could be turned on and off in QuPath v0.2.0-m2 software [13]. The prediction value (presence/absence) for submucosal invasion was not provided. The second evaluation was performed after a 4-week wash-out period. Diagnostic accuracy and mean diagnostic time were compared.

### Statistical methods

To evaluate the performance of the deep learning models developed in this study, the Dice Coefficient was calculated to assess the accuracy of tumor and muscularis mucosa segmentation at the patch level. The area under the receiver operating characteristic (AUROC) curve values were computed to measure the diagnostic accuracy of the model for detection of tumor and submucosal invasion at the WSI level. The optimal thresholds for sensitivity and specificity were determined using Youden’s J statistics.

Paired *t* tests were used to compare diagnostic times, with and without the assistance of the deep learning model. Statistical analyses were performed using SPSS 26.0 (IBM, Armonk, NY, USA), with a significance level of *P* < 0.05.

## Results

### Evaluation of tissue segmentation performance at the patch level

The performances of the tissue segmentation models were evaluated using patch-level data. The development set was split on a patient basis, with a 5:1 ratio between the training and internal validation sets, to ensure that the models were trained and evaluated on distinct patients. Three semantic segmentation models were assessed: U-Net++, DeepLabv3+, and SegFormer. Each model was tasked with segmenting the gastric tissue into three classes: tumor, muscularis mucosa, and others, from H&E-stained images.

Table 2 summarizes the performances of the models for different patch sizes and magnifications. The SegFormer consistently delivered the highest segmentation accuracy of the three models. It had the best performance with a patch size of 512 × 512 at 10 × magnification, achieving a mean Dice score of 0.826, 0.856 for tumor segmentation, and 0.797 for muscularis mucosa. Increasing the patch size or reducing the magnification generally improved performance, particularly for detection of tumors, which was likely to be due to the increased context provided in each patch.

**Table 2:** Tissue segmentation performance in terms of Dice coefficient using different semantic segmentation models and various patch sizes at different magnifications (Best results in bold)

Patch image size (magnification)	Classes	Model Architecture		
		Unet++	Deeplabv3++	Segformer
256 × 256 (20 ×)	Tumor	0.8232	0.8204	0.8243
	Muscularis mucosa	0.7712	0.7617	0.7778
	Average	0.7972	0.7911	0.8011
512 × 512 (20 ×)	Tumor	0.844	0.8399	0.848
	Muscularis mucosa	0.7864	0.7752	0.7891
	Average	0.8152	0.8076	0.8186
512 × 512 (10 ×)	Tumor	0.843	0.8492	**0.856**
	Muscularis mucosa	0.7842	0.7844	**0.7965**
	Average	0.8136	0.8168	**0.8263**

Upon reviewing the WSIs with tissue segmentation, we observed relatively poor performance in segmentation for poorly differentiated tumors compared to differentiated ones. To further investigate, we evaluated the tumor segmentation performance specifically for poorly differentiated tumors. In the internal validation set of 53 WSIs, seven WSIs with any poorly differentiated tumor components were identified. Among these, one WSI exhibited predominantly signet ring cell carcinoma, while six showed differentiated tumors with some poorly differentiated components. For these seven WSIs, the mean Dice score for tumor segmentation using SegFormer was 0.7886.

### Evaluation of the detection of tumor and submucosal invasion at the WSI level

Detection of tumor and submucosal invasion at the WSI level was evaluated using 111 WSIs from 61 patients, all of which were annotated by a pathologist for the presence of tumor and submucosal invasion. Tissue segmentation was performed using the SegFormer model, which demonstrated the best performance in the patch-level evaluation (Table 2). Empirically, *δ* is set to n^2^/2, and each grid is defined as 800 µm × 800 µm.

**Fig. 4:**
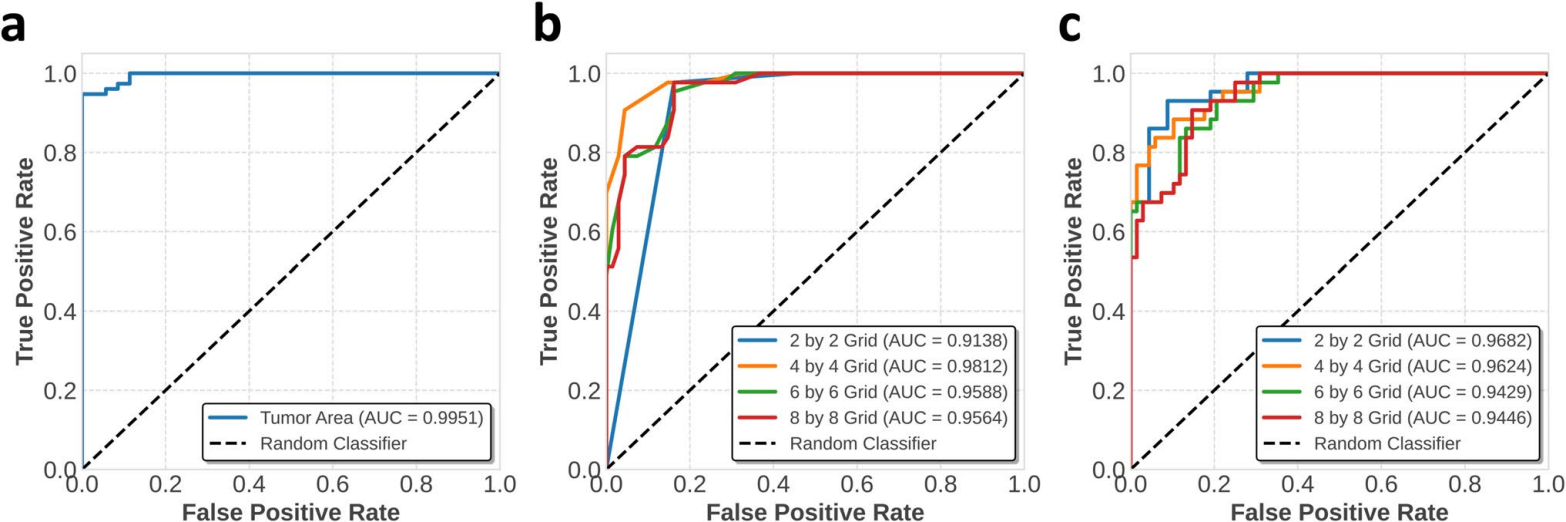
ROC AUC curve for the WSI-level detection of tumor and submucosal invasion. (**a**) ROC AUC curve for tumor detection at WSI-level, where the detected tumor area in a WSI is considered as the predicted probability, larger tumor area means higher probability that a WSI have tumor. (**b**) ROC AUC curve for submucosal invasion detection using the Max-based approach with different grid design at WSI-level. (**c**) ROC AUC curve for submucosal invasion detection using the mean-based approach with different grid design at WSI-level

The detected tumor area in each WSI was used as a predictor of the presence of tumor. A larger detected tumor area corresponded to a higher probability that the WSI contained a tumor. Figure 4a presents the AUROC curve for detection of tumor, where the method achieved an AUROC of 0.995, indicating high accuracy. After determining the optimal threshold using Youden’s J statistic, which maximizes the sum of sensitivity and specificity, the method had a specificity of 1.000, a sensitivity of 0.947, an F1 score of 0.973, a Matthews correlation coefficient (MCC) of 0.922, and an accuracy of 0.964, with a tumor area threshold set at 160,000 µm^2^ (Table 3).

**Table 3:** Evaluation results for WSI-level tumor detection and submucosal invasion detection (Max-based approach with 4 by 4 grid setting)

WSI-level	AUC	Specificity	Sensitivity	F1 score	MCC	Accuracy
Tumor Detection	0.9951	1.0000 (0.8857)	0.9474 (1.0000)	0.9730 (0.9743)	0.9221 (0.9173)	0.9640 (0.9640)
Submucosal Invasion Detection	0.9812	0.9559 (0.6765)	0.9070 (1.0000)	0.9176 (0.7963)	0.8667 (0.6690)	0.9369 (0.8018)

WSI, whole-slide image; AUC, area under the curve; MCC, Matthews correlation coefficient

Optimal threshold is selected by using Youden’s J statistic. (ㆍ) show results at 100% sensitivity using the corresponding threshold

For detection of submucosal invasion, Fig. 4b and c show the AUROC curves for the max-based and mean-based approaches, respectively. The max-based approach with a 4 × 4 grid setting achieved the best AUROC of 0.981. After determining the optimal threshold using Youden’s J statistic for this setting (Table 3), the method achieved a specificity of 0.956, a sensitivity of 0.907, an F1 score of 0.918, an MCC of 0.867, and an accuracy of 0.937, with a *p* threshold of 0.75.

### Diagnostic accuracy and interpretation times by pathologists

**Fig. 5:**
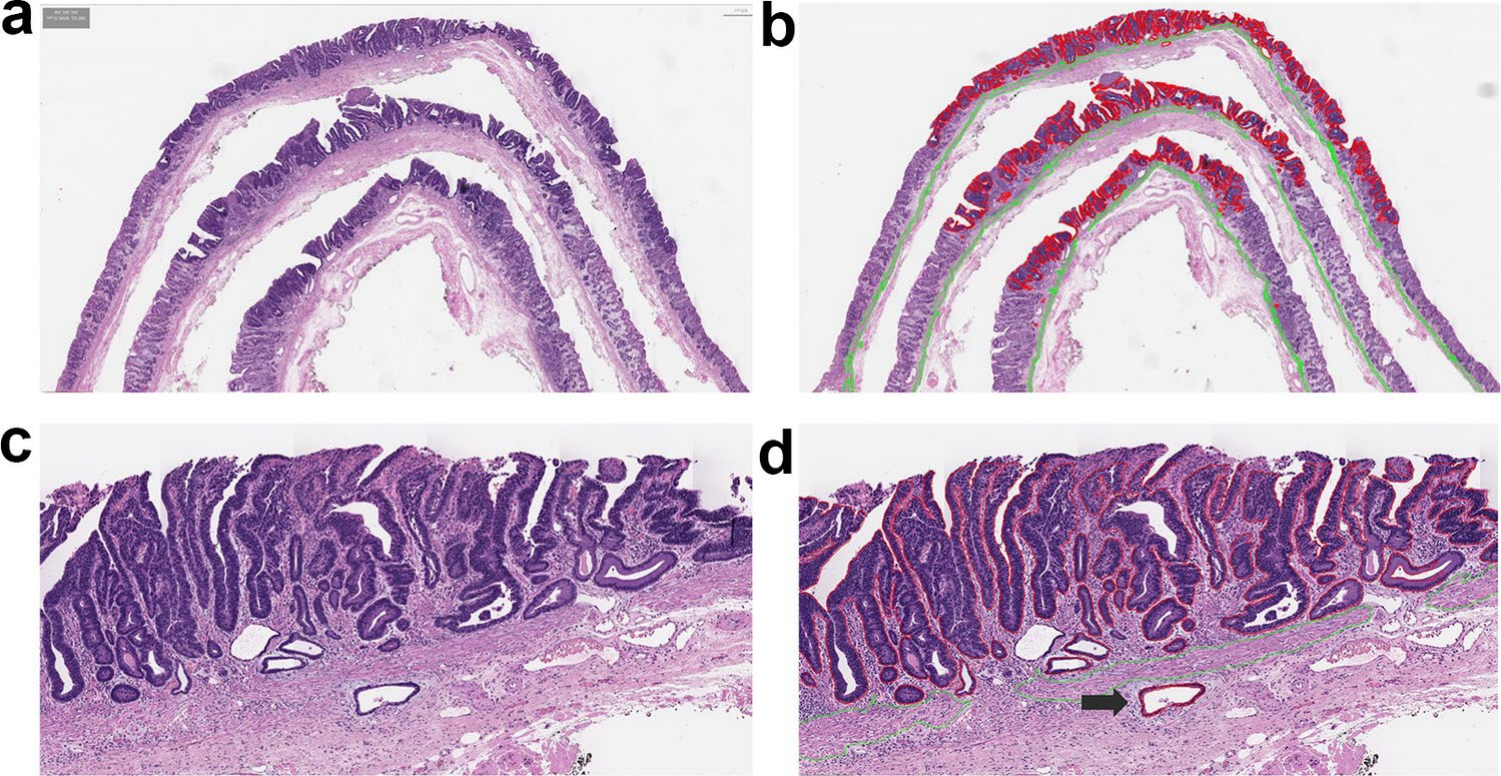
The representative image of (**a**, **b**) original WSI of ESD and (**c**, **d**) WSI with deep learning assistance. This model provides a heat-map flagging tumor (red) and muscularis mucosa (green) over the WSI. The model successfully flags subtle submucosal invasion (**d**, arrow)

Three general histopathologists, board-certified and practicing for 5–7 years, diagnosed 10 ESD specimens from digitally scanned images, unaided, and then re-evaluated them with the assistance of the deep learning model (Table 4). The three pathologists accurately detected the tumor area in the 10 specimens. There was no difference in tumor size, detection of lymphatic tumor emboli, or evaluation of lateral and deep resection margins, with and without the assistance of the deep learning model among all pathologists. However, the detection of submucosal invasion when assisted by the deep learning model varied among pathologists. While pathologist #3 had an improvement in accuracy in the detection of submucosal invasion (85.7% without assistance vs. 100% with assistance), pathologist #1 had a decrease in accuracy (100% without assistance vs. 85.7% with assistance). When that case was reviewed again, the diagnostic error was found to be due to the individual judgment error of the pathologist, not a fault in the deep learning model. The tumor cells just below the muscularis mucosa (depth: 54 µm) were highlighted by the deep learning model (Fig. 5). These results suggest that the deep learning-based diagnostic system supported accurate diagnosis and reduced the time required for pathologic diagnosis.

**Table 4:** Evaluation of three pathologists' performance with or without Deep Learning Model Assistance

	Unaided	Aided	*P* value
Accuracy for tumor size measurement			
Pathologist 1	100% (10/10)	100% (10/10)	
Pathologist 2	100% (10/10)	100% (10/10)	
Pathologist 3	100% (10/10)	100% (10/10)	
Accuracy for detecting submucosal invasion			
Pathologist 1	100% (7/7)	85.7% (6/7)	
Pathologist 2	85.7% (6/7)	85.7% (6/7)	
Pathologist 3	85.7% (6/7)	100% (7/7)	
Accuracy for margin evaluation			
Pathologist 1	100% (10/10)	100% (10/10)	
Pathologist 2	100% (10/10)	100% (10/10)	
Pathologist 3	100% (10/10)	100% (10/10)	
Accuracy for detecting lymphatic emboli			
Pathologist 1	100% (2/2)	100% (2/2)	
Pathologist 2	50% (1/2)	50% (1/2)	
Pathologist 3	50% (1/2)	50% (1/2)	
Mean reading time of each ESD case			
Overall	747 s (SD 339.27)	478 s (SD 228.39)	< 0.001
Pathologist 1	517 s (SD 123.9)	282 s (SD 62.459)	< 0.001
Pathologist 2	687 s (SD 258.85)	616 s(SD 253.19)	0.238
Pathologist 3	1036 s (SD 365.953)	537.3 s (179.66)	< 0.001

SD, Standard deviation

There was a significant difference in the overall evaluation time (747 s without assistance vs. 478 s with assistance, *P* < 0.001) (Table 4). Although pathologists #1 and #3 had a significant reduction in evaluation time, there was no difference for pathologist #2.

### Evaluation of tumor detection and submucosal invasion in surgically resected specimens

We tested our model on a preliminary set of 10 surgically resected early gastric cancers with submucosal invasion (SM1: *n* = 1, SM2: *n* = 2, SM3: *n* = 7). The model successfully detected tumors and submucosal invasion in all cases.

## Discussion

In recent years, deep learning models, particularly convolutional neural network technology, applied to the digitized WSIs of histopathology specimens, has led to remarkable progress in the identification and classification of images of various solid tumors, including breast [22] and prostate cancers [9, 23]. In this study, we developed and applied, for the first time, a deep learning-based diagnostic system for the histopathological diagnosis of gastric ESD specimens. This system reduces diagnosis time and has the potential to serve as a screening tool.

There have been several studies on deep learning-based histopathological diagnosis of gastric cancer, and recent studies have demonstrated high sensitivity and specificity in the classification of gastric cancer. [7, 8, 10–12, 24, 25] Song et al*.* developed a deep learning algorithm for accurate histopathological diagnosis of gastric cancer in biopsy specimens, and the model achieved almost 100% sensitivity and a mean specificity of 80.6%, in a real-world test [9, 25]. Park et al*.* developed a deep learning algorithm to divide WSIs from gastric biopsies into the following three groups: negative for dysplasia, tubular adenoma, or carcinoma; the mean AUROC was 0.979 for two-tier classification [8, 9]. The histopathological diagnosis of ESD specimens is substantially more complex than for diagnosis from biopsies. We developed and evaluated a deep learning model for diagnosing ESD specimens of EGC. The first objective was to train the model to correctly classify individual patches as tumor or muscularis mucosa. The model achieved Dice coefficients of 0.85 and 0.79 for segmentation of tumor and muscularis mucosa on the internal validation set, respectively. The Dice coefficient value observed in our study is comparable to those reported in other studies [26–28] and demonstrates reliable performance. It successfully detected tumor cells in most cases and demonstrated particularly high performance in detecting well-to-moderately differentiated tumors. However, it is limited for lesions with inherent difficulties, such as tumors with cautery artifacts, fragmented specimens, or poorly cohesive carcinoma. The model correctly classified the muscularis mucosa in most of the WSIs. It sometimes segmented the muscle component of the vascular structure but pathologists can easily distinguish it from muscularis mucosa.

The second objective was to determine whether the trained model could be used to effectively analyze WSIs with acceptable sensitivity and specificity. Since the Dice coefficient value alone is insufficient for clinical application, we evaluated the performance at the WSI level. For tumor, the method achieved very high specificity, sensitivity, and F1 score. ESD specimens usually yield slides containing tumor and non-tumor tissues, and scrutinizing all slides is time-consuming and requires a high level of concentration. The high specificity for tumor indicates that pathologists can examine slides classified as non-tumor more confidently and in less time. In this context, the assistance of the deep learning model can assist the pathologists to not only increase their diagnostic accuracy but to also undertake their busy workloads more rapidly and with greater confidence. The model had high sensitivity for tumor, particularly differentiated tumors. It appears to be useful when inexperienced pathologists have difficulty distinguishing between reactive atypia and well-differentiated foveolar type cancers. Furthermore, the detection of submucosal invasion is very important for the diagnosis of ESD specimens. The criteria for non-curative resection requiring further surgery includes submucosal invasion of ≥ 500 μm. In addition, lymphatic tumor emboli are frequently located in the submucosa. However, small foci of submucosal invasion or lymphatic emboli can easily be missed by inexperienced pathologists and may even be overlooked by specialist pathologists, particularly those with a heavy workload. The model achieved a high AUROC for detection of submucosal invasion, with high specificity, sensitivity, F1, MCC, and accuracy. The model successfully flagged subtle submucosal invasion (Fig. 5), indicating that it can alert pathologists. This study is a preliminary attempt at determining whether a deep learning algorithm can correctly recognize and classify complex diagnostic clinical ESD specimens, and the model shows encouraging potential toward deep learning-assisted diagnosis of ESD specimens. We suggest that this model has high potential to serve as a screening or assistance tool, not only for less experienced pathologists but also for specialists with increasing workloads. However, there are elements that cannot be replaced by deep learning algorithms. Contextual knowledge from pathologists remains essential in complex situations, such as detecting lymphatic tumor emboli or determining submucosal invasion in ulcerated or cautery-affected areas.

Finally, whether the developed model has a time-saving benefit or an accuracy improvement for pathologists in real practice was tested. Three general practicing histopathologists evaluated ESD specimens from 10 patients, with and without assistance from the deep learning model. The mean reading time per case was significantly shorter (747 s without assistance vs. 478 s with assistance). After the test, the pathologists commented that the assistance from the deep learning model was very useful as a screening tool. Although the performance of experienced pathologists were not tested in this study, previous studies have reported that less experienced pathologists tended to obtain greater improvement in accuracy with the assistance of a deep-model [6]. To evaluate improvement in accuracy, multiple factors must be evaluated in ESD diagnosis. Among them, there were no differences in tumor size, lymphatic emboli, or margin status. Meanwhile, the improvement in accuracy for identifying submucosal invasion varied among pathologists; one pathologist had a gain in sensitivity and submucosal invasion, and another pathologist missed lesions in an assisted setting. This emphasizes the importance of pathologist judgment in the process of diagnosing, even with the assistance of a deep learning model. While most studies reported that the assistance of a deep learning model improved the diagnostic accuracy of pathologists [6, 29–31], there were rare cases that were originally correct without assistance and then were incorrectly diagnosed in the assisted evaluation [31]. Ultimately, deep learning assistance did not enhance the accuracy of detecting lymphatic tumor emboli, which remains one of the most challenging and critical aspects of ESD diagnosis. This analysis underscores the importance of pathologists understanding the role and limitations of assistance tools, thereby ensuring active and meaningful participation in the diagnostic process [31].

This study had some limitations. First, all H&E stained slides were obtained from a single hospital; therefore, performance of the developed model has not been validated using an external cohort or alternative scanners. In addition, the number of WSIs was relatively small. However, a strength is the comprehensive pixel-level annotation with 2257 boxes of regions of interest and 83,839 patch images, performed by a gastrointestinal histopathologist. The annotation not only circled the area but also included precise marking of the glandular components of the tumors. Currently, the developed model has been built into software and is in the process of authorization. A multi-institutional study with a larger number of cases is being planned to validate its utility. Second, our algorithm has limitations in its application to poorly differentiated tumors. Currently, ESD is cautiously applied to poorly differentiated tumors only in cases of small size and endoscopically diagnosed mucosal cancer. Consequently, we were unable to include enough poorly differentiated tumors in the development cohort, which may have contributed to their suboptimal performance. Additional data on poorly differentiated tumors are required. Furthermore, for clinical applications, developing a deep learning model capable of diagnosing premalignant lesions is also essential. Finally, the performance of the test set at the WSI level was evaluated only by the presence or absence of evaluation indicators. No comparisons were made at the pixel level because of time constraints. Finally, the evaluation of pathologist performance was limited by the small number of patient samples. However, we plan to develop this diagnostic algorithm into software to test its usability across multiple institutions, along with the approval process.

We developed and evaluated a deep learning model to diagnose ESD specimens from patients with EGC. The developed model achieved satisfactory performance for segmentation of tumor and muscularis mucosa. At the WSI level, the model showed high accuracy in predicting tumor and submucosal invasion. The use of this model significantly shortened the mean time for the diagnosis of ESD specimens. This deep learning model can serve as a potential screening tool for histopathological diagnosis of ESD specimens.

## Data Availability

The datasets used and/or analyzed in the current study are available from the corresponding author upon reasonable request.
